# Whole system quality: local benchmarking to improve workforce planning

**DOI:** 10.1080/17571472.2016.1245241

**Published:** 2016-10-20

**Authors:** Deirdre Kelley-Patterson, Andy Knapton, Keith Hurst

**Affiliations:** a Centre for the Study of Policy and Practice in Health and Social Care, University of West London, Brentford, UK; b Strategic Modelling Analysis and Planning Limited (SMAP), Winchester, UK; c Hurst Research Ltd., Forrest Town, UK

**Keywords:** Workforce planning, benchmarking, primary care

## Why this matters to us

As a team of workforce analysts and academics with an interest in workforce planning, we are aware that the data available to support primary care workforce planning are disorganised and overwhelming. This makes it difficult for General Practice to extract meaningful and relevant information. We deliver workforce planning workshops across England. Participants at our workshops regularly express their frustration with the quantity of information they are required to produce and the quality of information they receive from other parts of the system. We are dismayed at what we sense to be growing cynicism with data generation and information analysis and are interested in stimulating a conversation about what data matter and how primary care teams can extract data that are useful.

## Key message

Bottom up ownership of information and local benchmarking is needed to underpin workforce planning in primary care.

## The case study: better benchmarking for primary care workforce planning in London

As with other parts of England, primary care in London faces considerable challenges in terms of recruiting and retaining staff. It is highly dependent upon locum, interim and agency staff. In London the challenge is less about recruitment of staff and more about retaining experienced, qualified staff.[1] As well as the operational challenges of sustaining service delivery, instability poses a threat to local learning about how to integrate care [2] and how to sustain partnerships for on-going collaborative improvements.[3] Understanding staffing variation across Clinical Commissioning Groups (CCGs) and identifying which areas perform well are important starting points for improved workforce planning.

In 2015 Health Education England London teams (HEE) commissioned the University of West London to deliver training for CCG and practice staff. One element of this support was the development of CCG regional profiles so that the eight CCGs of North West London (NWL) and the 12 in North Central and East London (NCEL) could benchmark workforce performance against one another and against average performance in England. We used one of the largest English healthcare databases available, the NHS benchmarking database, for this exercise. The database (available from the corresponding author) combines 1400 workforce planning and development data sets from around 20 sources into one easily accessible location. Organisations, localities and regions can use the database to benchmark their performance against best practice organisations (both national and local). This database has been used extensively in secondary care settings for over a decade. It clearly predicted one failing Trust’s demise in 2008 (though results were ignored) and has consistently demonstrated the link between an investment in staffing (and in particular in qualified staff) and mortality rates.

**Figure 1.**
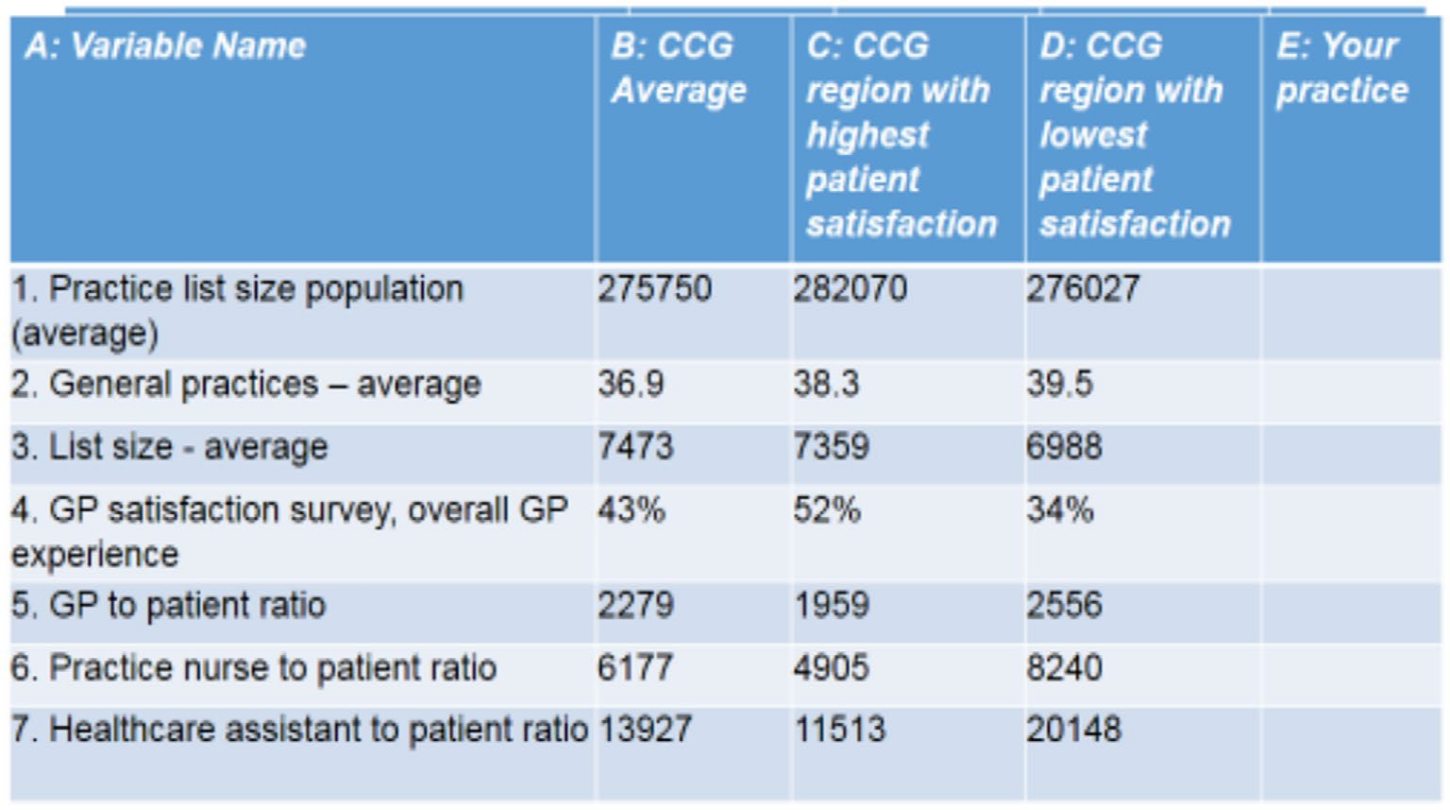
Is primary care service-quality related to staffing? FTE to list size ratios (Oct 2015).

We used this to help London CCGs and GP practices to consider the questions: *Is Primary Care service*-*quality related to staffing? How does my practice compare with ‘best practice sites?* The answers to these lie in Figure 1 below. Column B shows England averages, column C the results for the 16% of highest performing CCGs in England and Column C the averages for the poorest (16%) performers, as measured in terms of patient satisfaction. Unsurprisingly patients are more likely to be dissatisfied in those practices with high patient to GP ratios, high patient to practice nurse ratios and high patient to HCA ratios (rows 5, 6 and 7).

Figure 1 can be used to identify what ‘good looks like’ when assessing individual practice staffing. For example, how many practice nurses are needed if you want to model your workforce on that in best practice sites? If the practice list size is 7000 then divide this by the practice nurse to patient ratio in cell C6 to provide recommended full time equivalent (FTE) staffing numbers. (7000/4905 = 1.43 nurses). These data therefore provides an evidence base for estimating the number of staff needed to deliver a high-performing service, rather than a GP or practice manager having to guess. However the data don’t stand on its own and local people need to provide a narrative of what they mean. For example, a high patient to GP ratio may be because the practice cannot recruit or may have high turnover because of crime rates rather than as a consequence of a deliberate cost saving choice. We welcome further discussion around specifics of our methodology:

- Is patient satisfaction the best differentiating variable?
- Is London so different that a national average becomes unhelpful?

Better use of openly available strategic datasets will enable primary care to:

- Demonstrate many system relationships; for example the relationship between failing primary care services and ED admissions: - important evidence for a primary care community arguing for additional resources or different ways of working.
- Scrutinise and defend variation: The Carter report [4] states that the variation in people management practice across the NHS is holding back productivity improvement and that significant gains could be made by bringing the poorer performing organisations up to the level of the average. Whilst the focus of national attention is currently on secondary care, inevitably primary care performance will be scrutinised.
- Take ownership of decision-making at a local level. Primary care staff attending our workshops report that much of the data they are sent is used not for service and systems improvement but rather for micro management of performance. We concur with the views of Nigel Edwards of the Nuffield Trust that plans ‘to impose benchmarks from the top down risks turning into another round of the kneejerk centralisation that has served the NHS badly in recent years’.[5] Open resources to support local benchmarking and identify what is working well and where, are an essential ingredient of service improvement.

The Workforce Transformation team at HEE are developing a free, user-friendly interface for the NHS Benchmarking database to enable healthcare providers across the capital to benchmark what matters most to them. The trial version of this interface is available from Tom Houston, Healthy London Partnership at t.houston@nhs.net

This will be the first of a series of brief papers and in future editions we will identify where London staff really do provide ‘best practice’ care … and where there is room for improvement.

## Conflict of interest

All three authors deliver commercial workforce planning education and training courses commissioned by HEE for primary care organisations working across West, North, Central and East London. Keith Hurst develops and maintains the NHS Benchmarking Database and this tool is used to support a range of training and education programmes delivered by the University of West London

## Disclosure statement

No potential conflict of interest was reported by the authors.
